# Cardiovascular disease and risk of lung cancer incidence and mortality: A nationwide matched cohort study

**DOI:** 10.3389/fonc.2022.950971

**Published:** 2022-09-05

**Authors:** Ce Wang, Donghao Lu, Deirdre Cronin-Fenton, Chen Huang, Zeyan Liew, Dang Wei, Guoyou Qin, Yongfu Yu, Jiong Li

**Affiliations:** ^1^ Department of Biostatistics, School of Public Health, The Key Laboratory of Public Health Safety of the Ministry of Education, Fudan University, Shanghai, China; ^2^ Unit of Integrative Epidemiology, Institute of Environmental Medicine, Karolinska Institutet, Stockholm, Sweden; ^3^ Department of Epidemiology, Harvard T. H. Chan School of Public Health, Boston, MA, United States; ^4^ Department of Clinical Medicine–Department of Clinical Epidemiology, Aarhus University, Aarhus, Denmark; ^5^ Department of Environmental Health Sciences, Yale School of Public Health, New Haven, CT, United States; ^6^ Department of Global Public Health, Karolinska Institutet, Stockholm, Sweden; ^7^ Shanghai Institute of Infectious Disease and Biosecurity, Shanghai, China

**Keywords:** cardiovascular disease, lung cancer, incidence, mortality, survival

## Abstract

**Purpose:**

Previous studies have suggested a link between cardiovascular disease (CVD) and the subsequent development of lung cancer. However, empirical evidence on the association of CVDs, particularly type-specific CVDs, with lung cancer incidence and survival remains limited.

**Methods:**

The cohort study included 306,285 patients with CVD and 1,222,140 individuals without CVD. We performed stratified Cox regression to estimate the hazard ratio (HR).

**Results:**

During up to 42 years of follow-up, 243 (0.08%) and 537 (0.04%) participants were diagnosed with lung cancer among CVD patients and matched individuals, respectively. Patients with CVD had a 67% increased risk of lung cancer (HR: 1.67, 95% confidence interval [CI]: 1.42–1.96). The increased risks were observed in patients with heart disease (1.93, 1.30–2.85), vascular disease (1.88, 1.35–2.61), and hypertensive disease (1.46, 1.15–1.85), respectively. Patients with CVD had a 95% increased risk of lung cancer mortality (1.95, 1.50–2.55), particularly vascular disease (3.24, 1.74–6.02) and heart disease (2.29, 1.23–4.26). Patients with CVD diagnosed in middle adulthood (>40 years old) tended to have a higher incidence risk (3.44, 2.28–5.19) and mortality (3.67, 1.80–7.46) than those diagnosed at younger ages.

**Conclusions:**

Our findings on the association of CVD diagnosis, especially heart and vascular disease, with increased risk of lung cancer incidence and mortality suggest that CVD contributes to the development and worsening of lung cancer survival. In particular, people with CVD diagnosed in middle adulthood (>40 years old) would benefit from early preventive evaluation and screening for lung cancer.

## Introduction

Lung cancer is the most commonly diagnosed cancer, with 2.1 million new cases globally in 2018 (1). Despite advances in diagnosis and treatment, the five-year lung cancer survival has not improved significantly over the last several decades (2). Many lung cancer patients are not diagnosed until the advanced stages of the disease and have a low survival rate (3). Additionally, although tobacco use is still the most prevalent risk factor for lung cancer, approximately 25% of lung cancer cases occur in non-smokers (4). Accordingly, identifying high-risk groups besides smokers for prevention and early detection of lung cancer could be a new strategy to reduce morbidity and mortality.

Cardiovascular disease (CVD) is a leading contributor to the burden of disease (5, 6). Many shared risk factors, such as smoking, diabetes, obesity, CVD, and lung cancer, are often comorbid (5, 7). Previous studies have shown that inflammation, hypoxia, and clonal hematopoiesis play important roles in the association between CVD and cancer development (8). Several epidemiologic studies have suggested a link between CVD and the subsequent incidence of lung cancer (2, 9–15). However, those studies were mainly based on self-reported CVD, restricted to certain subtypes of CVDs, or had a relatively small sample size (2, 9–15). Age is an independent risk factor for both CVD and lung cancer, but no previous studies have evaluated the role of age at CVD diagnosis in the association. Finally, lung cancer remains the leading cause of cancer death, accounting for one-fifth of cancer-related deaths worldwide. However, few studies have investigated the association of overall and type-specific CVDs with subsequent lung cancer mortality (1, 13).

We hypothesized that CVD might contribute to lung cancer development and worsening survival (8). In this Danish population-based matched cohort study, we examined associations of overall and type-specific CVDs with lung cancer incidence and mortality and whether these associations varied by age at CVD diagnosis.

## Material and methods

### Study population

We conducted a population-based matched cohort study based on prospectively collected data from the Danish national registries (16). All live births and new residents in Denmark have been assigned a unique personal identification number (Central Personal Register number, CPR) since 1968, which allows accurate linkage of data from all registries (16). By Danish law, no informed consent is required for register-based studies based on anonymized data.

### CVD exposed cohort

We identified all patients with a first-time diagnosis (the index date) of CVD (n = 365,537) from the Danish National Patient Registry (DNPR, 1967–2006), and included them in the exposed cohort. Information on the diagnosis of CVD was coded using the International Classification of Disease (ICD) codes (ICD-8: 390–444.1, 444.3–458, 782.4; ICD-10: I00–I99) (**Table S1**). The DNPR includes hospital discharge diagnoses since 1977 and outpatient and emergency diagnoses from 1995 (16). CVD was defined as the first occurrence of CVD diagnosis in the DNPR, of cardiovascular drug use in the Danish National Prescription Registry, or of the cause of death as CVD in the Danish Register of Causes of Death. We also investigated the following CVD subtypes: heart disease (ICD-8: 393–398, 410–414, 420–429; ICD-10: I05–I09, I20–I28, I30–I52), hypertensive disease (ICD-8: 400–404; ICD-10: I10–I15), and vascular diseases (ICD-8: 430–438, 440–444.1, 444.3–448, 450–454, 456; ICD-10: I60–I83, I85–I87) (**Table S1**). We excluded 16,582 patients with CVD diagnosed before ten years old to exclude the most likely congenital heart diseases (17), and 2,315 with lung cancer before the diagnosis of CVD (**Figure S1**).

### The population-matched unexposed cohort

For each CVD patient, we randomly selected four individuals who were alive and CVD-free on the index date, and matched them, with replacement, on sex and age. The general population comparison cohort was selected using the same exclusion criteria as the CVD cohort (that is, no previous lung cancer diagnosis before the index date).

### The outcome of interest

The outcomes of interest were lung cancer incidence and mortality. Incident lung cancer was defined as the first diagnosis of lung cancer in the Danish Cancer Registry (DCR) and the DNPR, or death due to lung cancer in the Danish Causes of Death Register using ICD-codes (**Table S1**). The DCR systematically collected information on all incident cases of cancer in Denmark (16). The information on lung cancer mortality is from the Danish Causes of Death Register.

### Follow-up

Follow-up of all study participants was from the index date of CVD diagnosis until the first diagnosis of lung cancer (in the DCR, the DNPR, or Danish Causes of Death Register for lung cancer incidence, or in the Danish Causes of Death Register for lung cancer mortality), non-lung cancer death, emigration, or 31 December 2018, whichever came first.

### Covariates

Potential confounders were selected *a priori* based on previous knowledge (5). Confounders included obesity (yes, no), diabetes mellitus (yes, no), smoking disorders (yes, no), alcohol disorders (yes, no), cohabitation (yes, no, unknown), and education level (0–9, 10–14, ≥15 years, unknown). The covariate status was the latest status before the start of follow-up. Matching factors, including age and sex, were controlled by study design. Information on the diagnosis of obesity, smoking disorders, and alcohol disorders was retrieved from the DNPR, and diabetes mellitus from the Danish National Diabetes Register, the DNPR, and the Danish National Prescription Registry (16).

### Statistical analysis

We used competing risk analysis to estimate the cumulative incidence of lung cancer in the CVD cohort and matched unexposed cohort. Incidence rates were calculated per 1,000,000 person-years of follow-up. We performed stratified Cox proportional hazards regression to estimate the hazard ratio (HR) with 95% confidence intervals (CI) to assess the association between CVD and lung cancer with time since the index date as the time scale and matching factors (sex and age) as strata. Also, we examined the association of lung cancer with type-specific CVDs (heart disease, vascular disease, hypertensive disease, and other CVD diseases), age at CVD diagnosis (10–20, 21–30, 31–40, and ≥41 years) and effect modification by sex. We used multiple imputation to impute missing information (18).

We performed the following sensitivity analyses: First, to evaluate the impact of detection bias and possible overestimation of cancer risk following CVD diagnosis, we re-assessed the association between the CVD and risk of lung cancer incidence and applied different latency periods of 3 months, 6 months, 1 year, and 3 years between CVD diagnosis date and subsequent lung cancer diagnosis. That is, we restricted the follow-up to 3 months, 6 months, 1 year, or 3 years after the index date for both CVD patients and matched individuals (accordingly, any individuals diagnosed with lung cancer before these landmarks were excluded, and all individuals matched to excluded subjects were excluded as well). Second, as the DNPR was established in 1977, we could not accurately determine when the residents born before 1977 had the first diagnosis of CVD. We, therefore, examined the association between CVD and cancer by birth year (<1977 or ≥1997). Third, as smoking is a strong risk factor for lung cancer (19), but information on smoking was only recorded for pregnant women in the Danish Medical Birth Registry since 1991 (16), we identified a sub-cohort of female CVD patients registered in the Danish Medical Birth Registry as the exposed cohort, and we randomly sampled four CVD-free mothers from the birth register matched on age to the unexposed cohort (N = 546,370), and undertook stratified analysis by smoking status. Fourth, in order to evaluate the influence of uncontrolled confounding due to shared genetic or familial characteristics, we conducted sibship analysis by restricting offspring to sibling pairs born to the same mother irrespective of the father (half-sibling) (N = 236,716), and using the date of CVD diagnosis of the affected sibling as the index date for both siblings (20, 21). Fifth, the definition of CVD subtypes is broad in our study; we also assessed the association between specific CVD subtypes of heart disease and vascular disease with lung cancer incidence, such as ischemic heart disease and cerebrovascular disease. Sixth, atherosclerotic disease, the most likely associated with cancer risk, is unlikely to occur in the first three decades of life (22). We re-assessed the association of overall CVD and CVD subtypes with lung cancer incidence after excluding patients with CVD diagnosed up to the age of 30 years. All analyses were conducted using R Software (version 3.6.1).

## Results

This study included 306,285 CVD patients as the exposed cohort and 1,222,140 matched unexposed individuals (**Table S2**). The average age at diagnosis of CVD was 29.2±8.8 years, and approximately 55% of the study population were females. Exposed patients were likely to have a higher prevalence of a history of obesity, diabetes, smoking disorders, and alcohol disorders, compared with unexposed individuals (**Table S2**).

**Figure 1:**
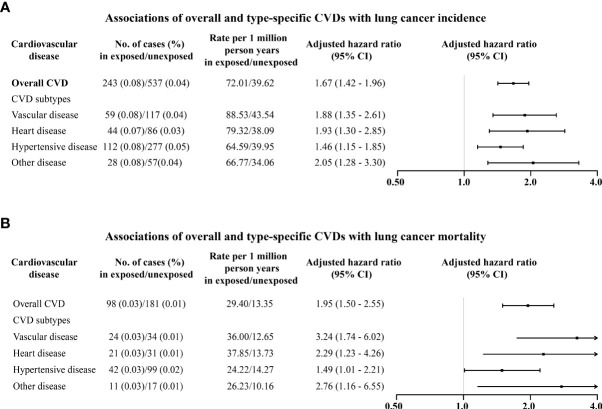
Associations of overall and type-specific CVDs with lung cancer incidence **(A)** and lung cancer mortality **(B)**. Controlled for matching factors (age and sex) by design and adjusted for obesity, diabetes mellitus, smoking disorders, alcohol disorders, marital status, and educational level.

During a follow-up of up to 42 years (median 10.2, IQR [5.5 to 16.1]), 243 (0.08%) and 537 (0.04%) were diagnosed with lung cancer among exposed patients and matched unexposed subjects, respectively (**Figure S2**). CVD patients had a 67% higher risk of lung cancer [HR: 1.67, 95% confidence interval (CI): 1.42–1.96] compared to their matched unexposed subjects (**Figure 1A**). The increased risks of lung cancer were also observed for vascular disease (1.88, 1.35–2.61), heart disease (1.93, 1.30–2.85), and hypertensive disease (1.46, 1.15–1.85) (**Figure 1A**).

**Figure 2:**
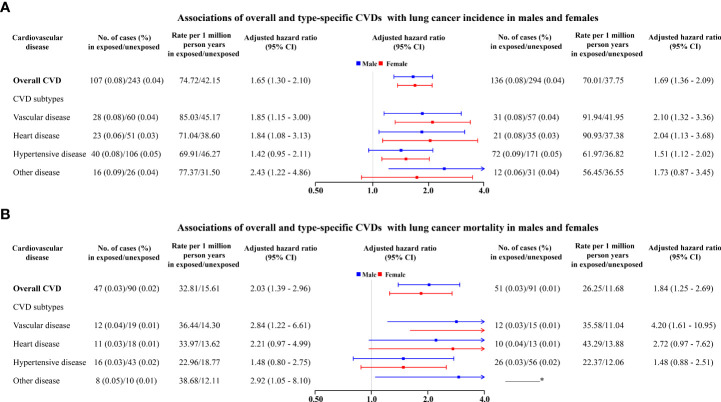
Associations of overall and type-specific CVDs with lung cancer incidence **(A)** and lung cancer mortality **(B)** in males (left) and females (right). ^*^ The number of deaths from lung cancer is less than 6, which is not allowed to report due to privacy protection. Controlled for matching factors (age and sex) by design and adjusted for obesity, diabetes mellitus, smoking disorders, alcohol disorders, marital status, and educational level.

Analyses stratified by sex found overall CVD and type-specific CVD were associated with increased risks of lung cancer among both males and females (**Figure 2A**). Regarding overall CVD, increased risks seemed slightly higher among females (1.69, 1.36–2.09) than in males (1.65, 1.30–2.10) but with overlapping CI. Similar patterns were also observed for heart disease, vascular disease, and hypertensive disease (**Figure 2A**).

**Figure 3:**
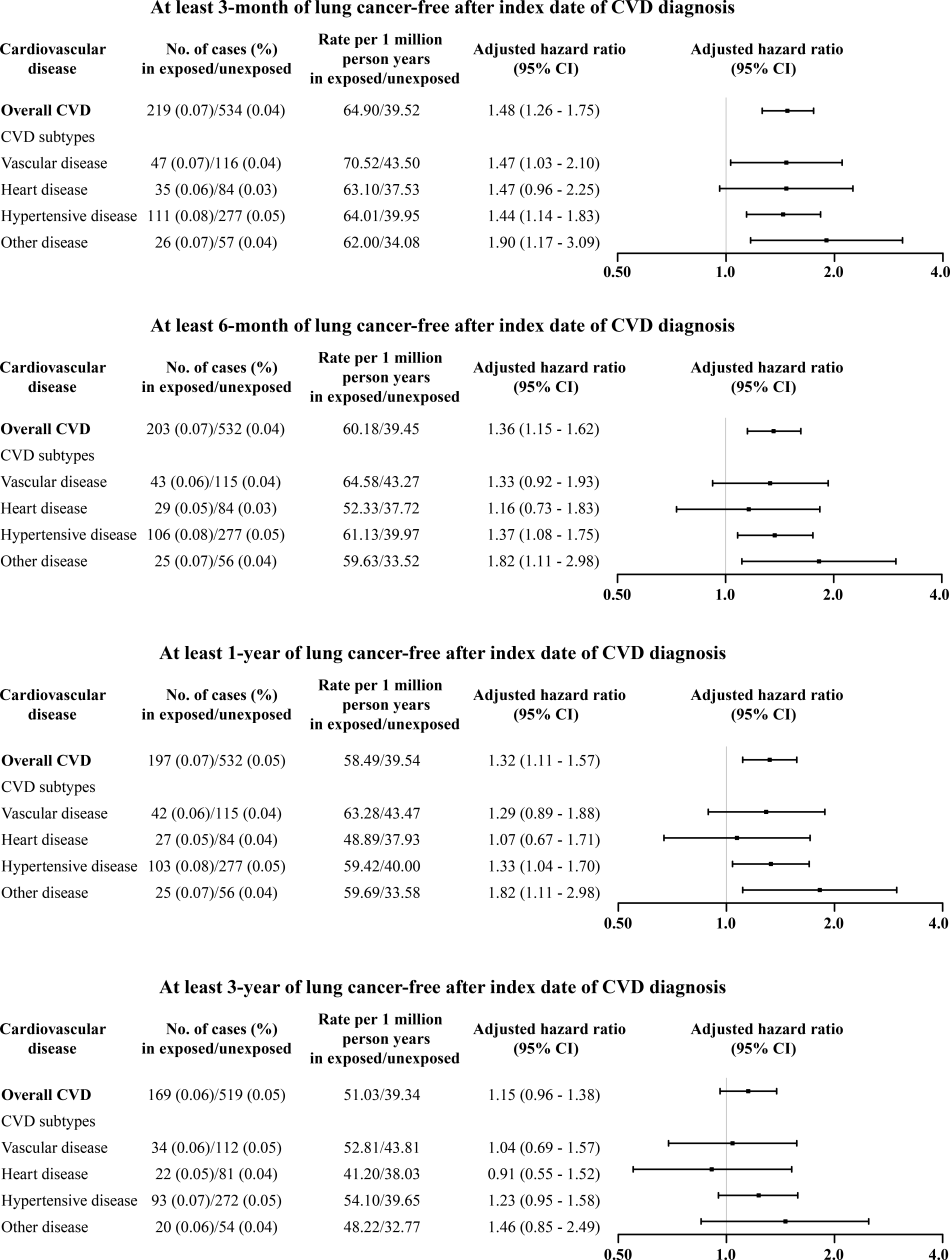
Associations of overall and type-specific CVDs with lung cancer incidence by different latency periods: **(A)** at least 3 months of lung cancer morbidity-free after index date; **(B)** at least 6 months of lung cancer-free after index date; **(C)** at least 1 year of lung cancer-free after index date; and **(D)** at least 3 years of lung cancer-free after index date. Controlled for matching factors (age and sex) by design and adjusted for obesity, diabetes mellitus, smoking disorders, alcohol disorders, marital status, and educational level. Cardiovascular disease and risk of lung cancer incidence and mortality: A nationwide matched cohort study.

The magnitude of the association between CVD and lung cancer incidence tended to increase with age at diagnosis of CVD (**Table 1**). Those who had been diagnosed with CVD during middle age (>40 years) tended to have higher lung cancer incidence (3.44, 2.28–5.19). The associations between CVD and lung cancer incidence attenuated yet may remain when latency periods were applied, namely, 3-month latency period (1.48, 1.26–1.75), 6-month latency period (1.36, 1.15–1.62), 1-year latency period (1.32, 1.11–1.57), and 3-year latency period (1.15, 0.96–1.38), respectively (**Figure 3**). Analyses restricted to those born after 1977 found an increased incidence of lung cancer after CVD diagnosis (3.00, 1.96–4.60) (**Table S3**). After adjusting for smoking status in the sub-cohort of pregnant women, the increased risk of lung cancer remained (1.33, 1.01–1.76) (**Table S4**). Further analyses stratified by smoking status found that the association of lung cancer with CVD was likely higher in non-smokers (1.49, 1.03–2.16) than in smokers (1.17, 0.78–1.77) (**Tables S4**, **S5**). The results from the sibling cohort also observed an increased risk of lung cancer incidence after CVD diagnosis (**Table S6**).

**Table 1:** Associations of overall CVD with lung cancer incidence by age at CVD diagnosis.

CVD diagnosisage	Exposure (CVD)	No. of cases (%)	Rate per 1million person-years	Crude hazard ratio(95% CI)^a^	Adjusted hazard ratio(95% CI)^b^
10–20					
	Non-exposed	49 (0.02)	15.70	1.0 (reference)	1.0 (reference)
	Exposed	15 (0.03)	19.17	1.03 (0.57–1.87)	1.02 (0.55–1.89)
21–30					
	Non-exposed	230 (0.05)	36.56	1.0 (reference)	1.0 (reference)
	Exposed	81 (0.07)	51.64	1.38 (1.07–1.78)	1.26 (0.97–1.66)
31–40					
	Non-exposed	203 (0.05)	57.05	1.0 (reference)	1.0 (reference)
	Exposed	98 (0.1)	111.31	1.99 (1.56–2.53)	1.83 (1.41–2.37)
>40					
	Non-exposed	55 (0.04)	94.58	1.0 (reference)	1.0 (reference)
	Exposed	49 (0.14)	342.38	3.60 (2.44–5.30)	3.44 (2.28–5.19)

^a^Controlled for matching factors (age and sex) by design.

^b^Controlled for matching factors (age and sex) by design and adjusted for obesity, diabetes mellitus, smoking disorders, alcohol disorders, marital status, and educational level.

Regarding lung cancer mortality, 98 (0.03%) and 181 (0.01%) died of lung cancer among exposed patients and matched unexposed subjects, respectively (**Figure S2**). In particular, CVD patients had a 95% higher risk of lung cancer mortality (1.95, 1.50–2.55) compared to matched unexposed subjects (**Figure 1B**), in particular for heart disease (2.29, 1.23–4.26) and vascular disease (3.24, 1.74–6.02). Increased mortality risks seemed to be higher among males (2.03, 1.39–2.96) than females (1.84, 1.25–2.69) (**Figure 2B**). The magnitude of the association between CVD and lung cancer mortality also tended to increase with age at diagnosis of CVD (**Table 2**).

**Table 2:** Associations of overall CVD with lung cancer mortality by age at CVD diagnosis.

CVD diagnosis age	Exposure (CVD)	No. of cases (%)	Rate per 1million person-years	Crude hazard ratio(95% CI)^a^	Adjusted hazard ratio(95% CI)^b^
10–30^*^					
	Non-exposed	77 (0.01)	8.18	1.0 (reference)	1.0 (reference)
	Exposed	43 (0.02)	18.29	2.19 (1.50–3.20)	2.01 (1.32–3.07)
31–40					
	Non-exposed	82 (0.02)	23.04	1.0 (reference)	1.0 (reference)
	Exposed	34 (0.03)	38.61	1.71 (1.14–2.55)	1.58 (1.03–2.42)
>40					
	Non-exposed	22 (0.02)	37.83	1.0 (reference)	1.0 (reference)
	Exposed	21 (0.06)	146.68	3.82 (2.10–6.94)	3.67 (1.80–7.46)

*Age groups 10–20 and 20–30 were combined as the number of events in age group 10–20 is less than 6, which is not allowed to report due to privacy protection.

^a^Controlled for matching factors (age and sex) by design.

^b^Controlled for matching factors (age and sex) by design and adjusted for obesity, diabetes mellitus, smoking disorders, alcohol disorders, marital status, and educational level.

## Discussion

In this population-based matched cohort study with up to 42 years follow-up, patients with CVD had a 67% increased risk of lung cancer and 95% increased risk of lung cancer mortality compared with those without CVD. Increased risks of lung cancer were found in subjects with heart disease and vascular disease, followed by hypertensive disease. Both lung cancer incidence and mortality tended to increase with increasing age at CVD diagnosis. The risk of lung cancer tended to increase after excluding CVD patients who remained lung cancer-free up to 3 years after their CVD diagnosis.

Taking advantage of a large sample size and long-term follow-up, we examined the association of overall CVD and CVD subtypes with the risk of lung cancer incidence and mortality. Our findings suggest a long-term impact of CVD on the risk of lung cancer, consistent with studies from Nordic and other European countries (2, 9–14). A Norwegian study including 97,087 participants found that self-reported CVD (a composite exposure including myocardial infarction, angina pectoris, or stroke) was associated with an increased occurrence of lung cancer in former and current smokers but not in never smokers (2). Except for the Norwegian study, other previous studies mainly focused on a single type-specific CVD (9–14). Several Danish studies have suggested an increased risk of lung cancer among patients with pulmonary embolism and superficial and deep venous thrombosis (10, 11). In addition, cohort studies from Finland, Sweden, Norway, and Austria suggested that high blood pressure was associated with an increased risk of lung cancer (12, 13). Also, an increased occurrence of lung cancer after vascular disease and chronic heart failure (HF) was observed in the Dutch population (standardized incidence ratios: 1.56, 1.31–1.83) and the Danish population (1.81, 1.54–2.12), respectively (9, 14). Compared with the 81% increased risk of lung cancer in the Danish study, such an association was not observed among the 28,341 Physicians’ Health Study male participants from the USA, which might be due to a relatively lower number of HF in the US study (1,420 HF) than in the Danish study (9,307 HF), or selection bias as the study participants were only male physicians with high socioeconomic status (15). Using a large sample from Danish registers, our study showed an association between CVD, including but not limited to some specific CVD subtypes, such as heart disease, vascular disease, and hypertensive disease, with lung cancer among both males and females, which extended the previous findings (2, 9–14). Furthermore, we first observed that the risk of lung cancer tended to increase with age at the diagnosis of CVD, especially after 40 years old. One possibility may be that aging contributes to a decline in immune function and affects various aspects of immune functional networks, thus increasing lung cancer risk in middle adulthood (23).

Empirical evidence is also limited for associating overall and type-specific CVDs with lung cancer survival, and has mainly focused on hypertension previously (13, 24–26). A UK study showed a higher proportion of lung cancer deaths, among people with CVD than among people without CVD (25). Also, studies from Norway, Austria, and Sweden found that high blood pressure levels were related to an increased mortality risk of lung cancer in males but not in females (13). In contrast, a Dutch study with a sample size of 11,075 participants reported that hypertensive women had a 2.19-fold (2.19, 1.21–3.97) risk of lung cancer mortality compared with women without hypertensive disorders (26). A Korean study found an association between hypertension and lung cancer mortality, but further analyses stratified by smoking status suggested that the increased risk was restricted to current smokers (24). Consistent with those findings, our observations suggested a higher risk of lung cancer mortality after a diagnosis of CVD, including heart disease, vascular disease, and hypertensive disease, although most of the CIs are crossing the unity due to a small number of events.

The potential mechanisms underlying the association between CVD and increased incidence and mortality of lung cancer remain unclear (8). Inflammation has been proposed to explain the association between CVD and lung cancer (8). Most CVDs develop from atherosclerosis, which begins with damaged endothelial cells allowing cholesterol-containing low-density lipoprotein particles to accumulate and oxidize in the vessel walls (27). These processes trigger a chronic inflammatory response and the overexpression of proinflammatory factors, especially interleukin-1β (8, 27). Tumor-promoting inflammation would block anti-tumor immunity and active inflammatory responses in the tumor microenvironment to promote cancer development (28). Studies have shown that canakinumab, a potent interleukin-1β inhibitor that would reduce cardiovascular events in treated participants, could significantly reduce lung cancer incidence and is beneficial for lung cancer survival (8, 29, 30).

### Strengths and limitations

Our study has several strengths. First, the diagnoses of CVD and lung cancer were obtained prospectively and independently, minimizing the probability of recall and selection bias (16). Second, the completeness and validity of the Danish Cancer Registry have been reported to be high (31). Third, the large sample size and long follow-up of the cohort allowed us to assess the association between subtype CVDs and lung cancer.

Several limitations need to be noted. First, although the Danish registers allowed us to adjust for a wide range of confounders, we cannot omit the possibility of residual confounding by some uncontrolled factors. Smoking is the leading risk factor for lung cancer, and a lack of information on smoking may partially account for the association between a diagnosis of CVD and a subsequent risk of lung cancer. We have adjusted for smoking disorders, which may have controlled for the impact of smoking to some degree. In addition, as the smoking status was recorded for pregnant women in the Danish Medical Birth Registry, analysis adjusting for smoking status in the sub-cohort of female CVD patients and the unexposed cohort suggested an increased risk of lung cancer after a diagnosis of CVD. Accordingly, these findings suggest that the observed associations are less likely completely attributable to confounding by smoking. Moreover, our sibship design yielded results similar to those of the main analysis in the matched population cohort. Second, potential misclassification bias might remain because subjects born before 1977 with a potential CVD event would be misclassified as unexposed subjects. Also, individuals born between 1967 and 1977 might have a first CVD diagnosis before 1977 and a second CVD diagnosis later in life, which would lead to an underestimation of follow-up time at risk. However, the association persisted in sensitivity analyses restricted to subjects born after 1977. Third, patients with CVD might have a higher possibility of being detected as having cancer due to their more frequent visits to doctors than the general population, leading to detection bias. However, we repeated our main analysis with a 1-year latency period and a 3-year latency period between CVD diagnosis and cancer event, and the risk of lung cancer tended to be increased using a 3-year latency period. Fourth, the maximum CVD diagnosis age was 52 years old; therefore, our findings might not be generalized to people with CVD diagnosed at later ages. Fifth, the definitions of heart disease and vascular disease are too broad in our study, meaning that not all specific heart disease or vascular disease is associated with lung cancer. Thus, the association of specific heart disease or vascular disease with lung cancer requires further research.

## Conclusion

Our findings suggest that patients with CVD, especially heart and vascular disease, have increased lung cancer incidence and mortality risks. CVD may be an independent risk factor for lung cancer, and people with CVD in middle adulthood may benefit more from lung cancer screening than those diagnosed at younger ages.

## Data availability statement

The data analyzed in this study is subject to the following licenses/restrictions: All data is stored at the secure platform of Denmark Statistics, which is the central authority on Danish statistics with the mission to collect, compile and publish statistics on the Danish society. Due to restrictions related to Danish law and protecting patient privacy, the combined set of data as used in this study can only be made available through a trusted third party, Statistics Denmark (https://www.dst.dk/en/kontakt). This state organization holds the data used for this study. University-based Danish scientific organizations can be authorized to work with data within Statistics Denmark and such organization can provide access to individual scientists. Researchers can apply for access to these data when the request is approved by the Danish Data Protection Agency: https://www.datatilsynet.dk, the email address for the Danish Data Protection Agency is: dt@datatilsynet.dk. Requests for data may be sent to Statistics Denmark: http://www.dst.dk/en/OmDS/organisation/TelefonbogOrg.aspx?kontor=13&tlfbogsort=sektion or the Danish Data Protection Agency: https://www.datatilsynet.dk.

## Ethics statement

The study was approved by the Data Protection Agency (Record No. 2013-41-2569). By Danish law, no informed consent is required for a register-based study based on anonymized data.

## Author contributions

CW, GQ, YY, and JL conceived and designed the study. YY undertook the statistical analysis. CW and CH drafted the manuscript. All authors provided critical input to the analyses. All authors interpreted the data and revised the manuscript critically. YY and JL had full access to all the data in the study and all authors had final responsibility for the decision to submit for publication. All authors contributed to the article and approved the submitted version.

## Funding

This work was supported by the Independent Research Fund Denmark (grant numbers DFF-6110-00019B, 9039-00010B, and 1030-00012B), the Nordic Cancer Union (R275-A15770, R278-A15877), the Karen Elise Jensens Fond (2016), the Novo Nordisk Fonden (NNF18OC0052029), the Shanghai Rising-Star Program (21QA1401300), the National Natural Science Foundation of China (Nos. 82073570 and 82173612), the Shanghai Municipal Natural Science Foundation (22ZR1414900), and the Shanghai Municipal Science and Technology Major Project (ZD2021CY001). The sponsors had no role in study design, data collection, data analysis, data interpretation, or writing of this report.

## Conflict of interest

The authors declare that the research was conducted in the absence of any commercial or financial relationships that could be construed as a potential conflict of interest.

## Publisher’s note

All claims expressed in this article are solely those of the authors and do not necessarily represent those of their affiliated organizations, or those of the publisher, the editors and the reviewers. Any product that may be evaluated in this article, or claim that may be made by its manufacturer, is not guaranteed or endorsed by the publisher.
