# Supplementary material for: Cardiovascular disease and risk of lung cancer incidence and mortality: A nationwide matched cohort study
Source: Front Oncol. 2022 Sep 5;12:950971. doi: 10.3389/fonc.2022.950971 (PMC9486704; doi:10.3389/fonc.2022.950971)
Supplement: Supplementary file 1 [file DataSheet_1.docx]

**Cardiovascular disease and risk of lung cancer incidence and mortality: A nationwide matched cohort study**

**Supplementary Material**

**Table of contents**

Table S1. Definition of cardiovascular disease, lung cancer and covariates

Table S2. Characteristics of CVD cohort and matched unexposed cohort.

Table S3. Association of overall CVD with lung cancer incidence and mortality by birth year.

Table S4. Association of overall CVD lung cancer with lung cancer incidence in a sub-cohort of pregnant women with available information on smoking (N= 546,370)

Table S5. Characteristics of CVD cohort and matched general population comparison cohort in the sub-cohort.

Table S6. Association of overall CVD with lung cancer incidence in sibling cohort (N=236,716)

Table S7 Association of specific CVD subtypes of heart disease and vascular disease with lung cancer incidence

Table S8. Association of overall CVD and type-specific CVDs with lung cancer incidence in patients with CVD diagnosed over the age of 30

Figure S1. Flow chart of study population

Figure S2 Cumulative incidence of lung cancer incidence and mortality in CVD cohort (exposed) and matched unexposed cohort

**Table S1. Definition of cardiovascular disease, lung cancer and covariates.**

| Diagnoses | ICD-7 | ICD-8 | ICD-10 | ATC |
| --- | --- | --- | --- | --- |
| **Overall CVD** |  | 390-444.1, 444.3-458, 782.4 | I00-I99 |  |
| **Heart disease** |  | 393-398, 410-414, 420-429 | I05-I09, I20-I28, I30-I52 |  |
| Rheumatic heart disease |  | 393-398 | I05-I09 |  |
| Ischemic heart diseases |  | 410-414 | I20-I25 |  |
| [Cardiomyopathy](https://en.wikipedia.org/wiki/Cardiomyopathy) |  | 425 | I42-I43 |  |
| Heart Failure |  | 427.0,427.1,782.4 | I110, I130, I132, I50 |  |
| Other heart disease |  | Remainder of codes (393-398, 410-414, 420-429) | Remainder of codes (I05-I09, I20-I28, I30-I52) |  |
| **Vascular disease** |  | 430-438, 440-444.1, 444.3-448, 450-454, 456 | I60-I83, I85-I87 |  |
| Cerebrovascular disease |  | 430-438 | I60-I69 |  |
| Arterial disease |  | 440-444.1,444.3,446-447 | I70-I77 |  |
| Arteriosclerosis |  | 440 | I70 |  |
| Aortic aneurysm |  | 441 | I71 |  |
| Other arterial disease |  | Remainder of codes (440-444.1,444.3,446-447) | Remainder of codes (I70-I77) |  |
| Vein disease |  | 450-454,456 | I80-I83, I85-I87 |  |
| Superficial thrombophlebitis |  | 451.01 | I80.0 |  |
| Deep venous thrombosis |  | 451 | I80.1–I80.3 |  |
| Other vein disease |  | Remainder of codes (450-454,456) | Remainder of codes (I80-I83, I85-I87) |  |
| Other vascular disease |  | Remainder of codes (430-438, 440-444.1, 444.3-448, 450-454, 456) | Remainder of codes (I60-I83, I85-I87) |  |
| **Hypertensive disease** |  | 400-404 | I10-I15 | See specified definition^a^ |
| **Other CVDs** |  | Remainder of codes (390-444.1, 444.3-458, 782.4) | Remainder of codes (I00-I99) |  |
| **Outcome** |  |  |  |  |
| Lung cancer | 162 | 162 | C33, C34 |  |
| **Baseline covariates** |  |  |  |  |
| Obesity |  | 277 | E66 |  |
| Diabetes mellitus |  | 249, 250, 63474 | E10, E11, O24, Y6449 | A10A, A10B |
| Smoking disorder |  | 490-492 | F17.2, J40-J44, T65.2, Z57.31, Z71.6, Z72.0, Z77.22 |  |
| Alcohol disorders |  | 291, 303, 305.0, 425.5, 535.3 | F10.0-F10.9, I42.6, G31.2, G62.1, G72.1, K29.2, K70.0-K70.4, K70.9, K85.2, K86.0, Q86.0 |  |

^a^ We identified subjects with hypertension from combination treatment with at least two of the following classes of antihypertensive drugs:

I· Alpha adrenergic blockers (C02A, C02B, C02C)

II· Non-loop diuretics (C02DA, C02L, C03A, C03B, C03D, C03E, C03X, C07C, C07D, C08G, C09BA, C09DA, C09XA52)

III· Vasodilators (C02DB, C02DD, C02DG, C04, C05)

IV· Beta blockers (C07)

V· Calcium channel blockers (C07F, C08, C09BB, C09DB)

VI· Renin-angiotensin system inhibitors (C09)

**Table S2. Characteristics of CVD cohort and matched unexposed cohort.**

|  | CVD cohort  (N= 306285) | Matched unexposed cohort (N=1222140) |
| --- | --- | --- |
| Age, Mean (SD) | 29.19 (8.82) | 29.19 (8.82) |
| Sex, No.(%) |  |  |
| Male | 137503 (44.9) | 550012 (44.9) |
| Female | 168782 (55.1) | 675128 (55.1) |
| Birth year, No.(%) |  |  |
| $<$1977 | 163670 (53.4) | 654680 (53.4) |
| 1977~1986 | 92277 (30.1) | 369108 (30.1) |
| 1987~1996 | 41410 (13.5) | 165640 (13.5) |
| 1997~2006 | 8923 (2.9) | 35712 (2.9) |
| Education (years), No.(%) |  |  |
| 0~9 | 99514 (32.5) | 268774 (21.9) |
| 10~14 | 135230 (44.2) | 456646 (37.3) |
| ~15 | 48695 (15.9) | 217636 (17.8) |
| Unknown | 22846 (7.5) | 282084 (23.0) |
| Cohabitation status, No.(%) |  |  |
| Yes | 97312 (31.8) | 344252 (28.1) |
| No | 133004 (43.4) | 452231 (36.9) |
| Unknown | 75969 (24.8) | 428657 (35.0) |
| Diabetes mellitus, No.(%) |  |  |
| Yes | 11137 (3.6) | 14760 (1.2) |
| No | 295148 (96.4) | 1210380 (98.8) |
| Obesity, No.(%) |  |  |
| Yes | 26675 (8.7) | 45596 (3.7) |
| No | 279610 (91.3) | 1179544 (96.3) |
| Smoking disorders, No.(%) |  |  |
| Yes | 4118 (1.3) | 4824 (0.4) |
| No | 302167 (98.7) | 1220316 (99.6) |
| Alcohol disorders, No.(%) |  |  |
| Yes | 6027 (2.0) | 10039 (0.8) |
| No | 300258 (98.0) | 1215101 (99.2) |

**Table S3. Association of overall CVD with lung cancer incidence by birth year.**

| Birth year | Exposure (CVD) | No. of cases (%) | Rate per 1 million person-years | Crude hazard ratio  (95% CI)^a^ | Adjusted hazard ratio  (95% CI)^b^ |
| --- | --- | --- | --- | --- | --- |
| <1977 |  |  |  |  |  |
|  | Non-exposed | 483 (0.07) | 58.30 | 1.0 (reference) | 1.0 (reference) |
|  | Exposed | 202 (0.14) | 97.70 | 1.65(1.40 - 1.95) | 1.53(1.29 - 1.82) |
| ≥1977 |  |  |  |  |  |
|  | Non-exposed | 54 (0.01) | 10.25 | 1.0 (reference) | 1.0 (reference) |
|  | Exposed | 41 (0.03) | 31.37 | 3.09(2.06 - 4.65) | 3.00(1.96 - 4.6) |

^a^ Controlled for matching factors (age and sex) by design

^b^ Controlled for matching factors (age and sex) by design and adjusted for obesity, diabetes mellitus, smoking disorders, alcohol disorders, marital status, and educational level

**Table S4. Association of overall CVD with lung cancer incidence in a sub-cohort of pregnant women with available information on smoking (N= 546,370).**

| Smoking | Exposure (CVD) | No. of cases (%) | Rate per 1 million person-years | Crude Hazard ratio  (95% CI)^a^ | Adjusted Hazard ratio  (95% CI)^b^ |
| --- | --- | --- | --- | --- | --- |
| Overall |  |  |  |  |  |
|  | Non-exposed | 197 (0.05) | 37.22 | reference | reference |
|  | Exposed | 70 (0.06) | 53.00 | 1.42 (1.08 - 1.87) | 1.33 (1.01 - 1.76) |
| Smoking |  |  |  |  |  |
|  | Non-exposed | 87 (0.10) | 100.96 | reference | reference |
|  | Exposed | 32 (0.10) | 119.13 | 1.17 (0.78 - 1.75) | 1.17 (0.78 - 1.77) |
| Non-smoking |  |  |  |  |  |
|  | Non-exposed | 110 (0.03) | 24.83 | reference | reference |
|  | Exposed | 38 (0.04) | 36.12 | 1.46 (1.01 - 2.11) | 1.49 (1.03 - 2.16) |

^a^ Controlled for matching factors (age) by design

^b^ Controlled for matching factors (age) by design and adjusted for obesity, diabetes mellitus, smoking disorders, alcohol disorders, marital status, and educational level

**Table S5. Characteristics of CVD cohort and matched general population comparison cohort in the sub-cohort of pregnant women.**

|  | CVD cohort  (N= 109274) | Matched unexposed cohort (N= 437096) |
| --- | --- | --- |
| Age, Mean (SD) | 28.91 (7.63) | 28.91 (7.63) |
| Birth year, No.(%) |  |  |
| $<$1977 | 55753 (51.0) | 223012 (51.0) |
| 1977~1986 | 43385 (39.7) | 173540 (39.7) |
| 1987~1996 | 10048 (9.2) | 40192 (9.2) |
| 1997~2006 | 88 (0.1) | 352(0.1) |
| Education (years), No.(%) |  |  |
| 0~9 | 31530 (28.9) | 94288 (21.6) |
| 10~14 | 50433 (46.2) | 191685 (43.9) |
| ~15 | 23260 (21.3) | 112252 (25.7) |
| Unknown | 4051 (3.7) | 38871 (8.9) |
| Cohabitation status, No.(%) |  |  |
| Yes | 42620 (39.0) | 162318 (37.1) |
| No | 33148 (30.3) | 127130 (29.1) |
| Unknown | 33506 (30.7) | 147648 (33.8) |
| Diabetes, No.(%) |  |  |
| Yes | 4556 (4.2) | 11832 (2.7) |
| No | 104718 (95.8) | 425264 (97.3) |
| Obesity, No.(%) |  |  |
| Yes | 16094 (14.7) | 44477 (10.2) |
| No | 93180 (85.3) | 392619 (89.8) |
| Smoking disorders, No.(%) |  |  |
| Yes | 1468 (1.3) | 2561 (0.6) |
| No | 107806 (98.7) | 434535 (99.6) |
| Smoking Status, No.(%) |  |  |
| Yes | 21466 (19.6) | 69680 (15.9) |
| No | 87808 (80.4) | 367416 (84.1) |
| Alcohol disorders No.(%) |  |  |
| Yes | 1230 (1.1) | 2759 (0.6) |
| No | 108044 (98.9) | 434337 (99.4) |

Table S6. Association of overall CVD with lung cancer incidence in sibling cohort (N=236,716)

| Exposure (CVD) | No. of cases (%) | Rate per 1 million person-years | Crude Hazard ratio  (95% CI) | Adjusted Hazard ratio  (95% CI)^a^ |
| --- | --- | --- | --- | --- |
|  |  |  |  |  |
| Non-exposed | 9 (0.006) | 7.01 | reference | reference |
| Exposed | 22 (0.022) | 23.82 | 4.13(1.81 - 9.43) | 6.67(1.57 - 28.42) |

^a^ Adjusted for birth year, sex, maternal education level, parity

**Table S7 Association of specific CVD subtypes of heart disease and vascular disease with lung cancer incidence**

| **Exposure ^a^** | Case (Rate*) | Crude HR^b^ | Adjusted HR^c^ |
| --- | --- | --- | --- |
| **Heart disease** |  |  |  |
| Ischemic heart diseases | 9(95.46)/17(44.55) | 2.20(0.97-4.98) | 1.67(0.65-4.30) |
| Other heart disease | 31(71.64)/67(38.18) | 1.79(1.16-2.75) | 1.83(1.16-2.89) |
| **Vascular disease** |  |  |  |
| **Cerebrovascular disease** | 15(138.30)/22(48.11) | 3.01(1.53-5.95) | 3.30(1.47-7.41) |
| **Arterial disease** | 7(109.30)/12(46.49) | 2.13(0.83-5.47) | 2.25(0.77-6.51) |
| Other arterial disease | 6(119.57)/9(44.67) | 2.38(0.84-6.80) | 2.43(0.73-8.09) |
| **Vein disease** | 37(80.97)/79(43.31) | 1.83(1.23-2.71) | 1.83(1.21-2.76) |
| Deep venous thrombosis | 10(120.44)/13(38.49) | 3.29(1.42-7.62) | 2.83(1.09-7.32) |
| Other vein disease | 25(69.34)/63(44.00) | 1.51(0.94-2.41) | 1.60(0.98-2.60) |

* Per 1,000,000 person years

^a^ The number of events is less than 6 in some CVD subtype, which is not allowed to report due to privacy protection

^b^ Controlled for matching factors (age and sex) by design

^c^ Controlled for matching factors (age and sex) by design and adjusted for obesity, diabetes mellitus, smoking disorders, alcohol disorders, marital status, and educational level

**Table S8. Association of overall CVD and type-specific CVDs with lung cancer incidence in patients with CVD diagnosed over the age of 30**

| **Exposure** | Case (Rate^*^) | Crude HR^a^ | Adjusted HR^b^ |
| --- | --- | --- | --- |
| **Overall CVD** | 147(143.61)/258(62.32) | 2.33(1.90-2.86) | 2.16(1.74-2.68) |
| **Heart disease** | 32(183.63)/46(64.53) | 2.89(1.83-4.55) | 2.95(1.80-4.86) |
| **Vascular disease** | 36(150.25)/54(55.28) | 2.74(1.79-4.19) | 2.45(1.55-3.87) |
| **Hypertensive disease** | 59(122.13)/132(68.03) | 1.80(1.32-2.45) | 1.65(1.18-2.31） |
| **Other CVD disease** | 20(157.96)/26(51.00) | 3.33(1.84-6.03) | 3.38(1.81-6.33) |

^*^Per 1,000,000 person years

^a^ Controlled for matching factors (age and sex) by design

^b^ Controlled for matching factors (age and sex) by design and adjusted for obesity, diabetes mellitus, smoking disorders, alcohol disorders, marital status, and educational level

**
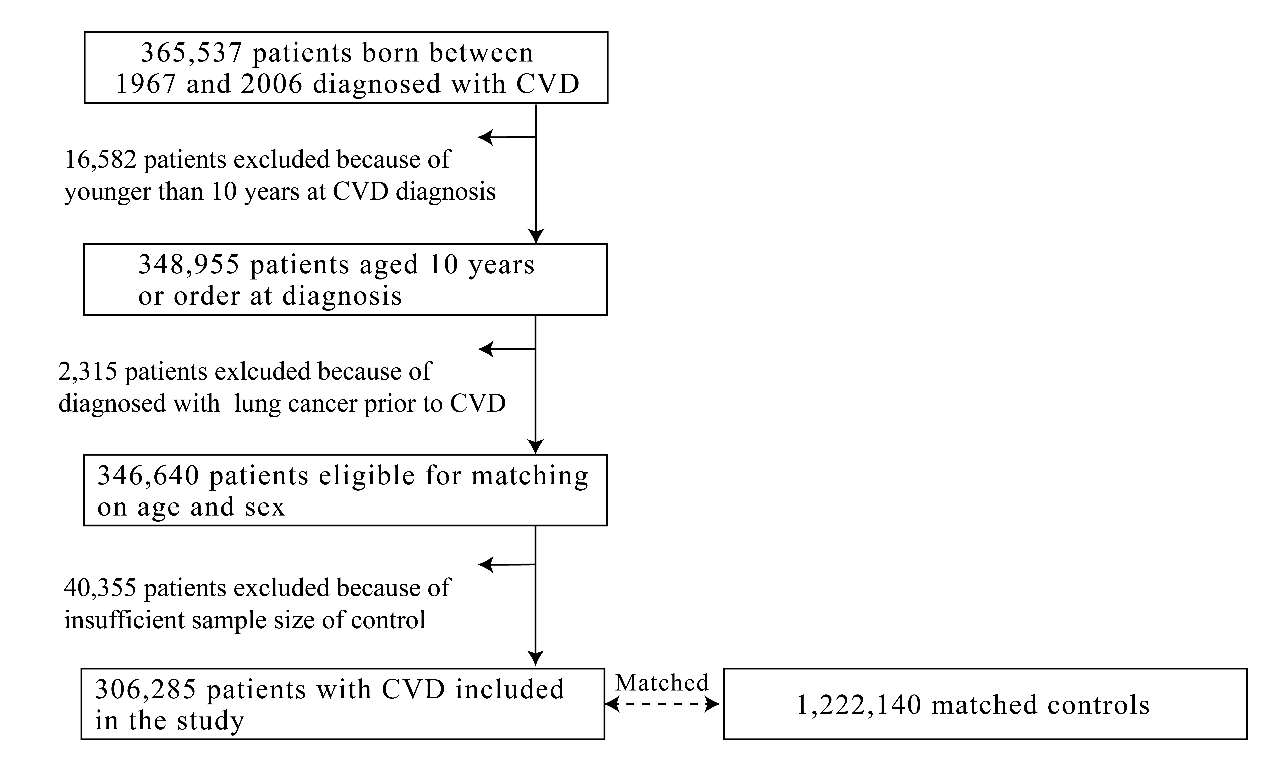
Figure S1. Flow chart of study population.**


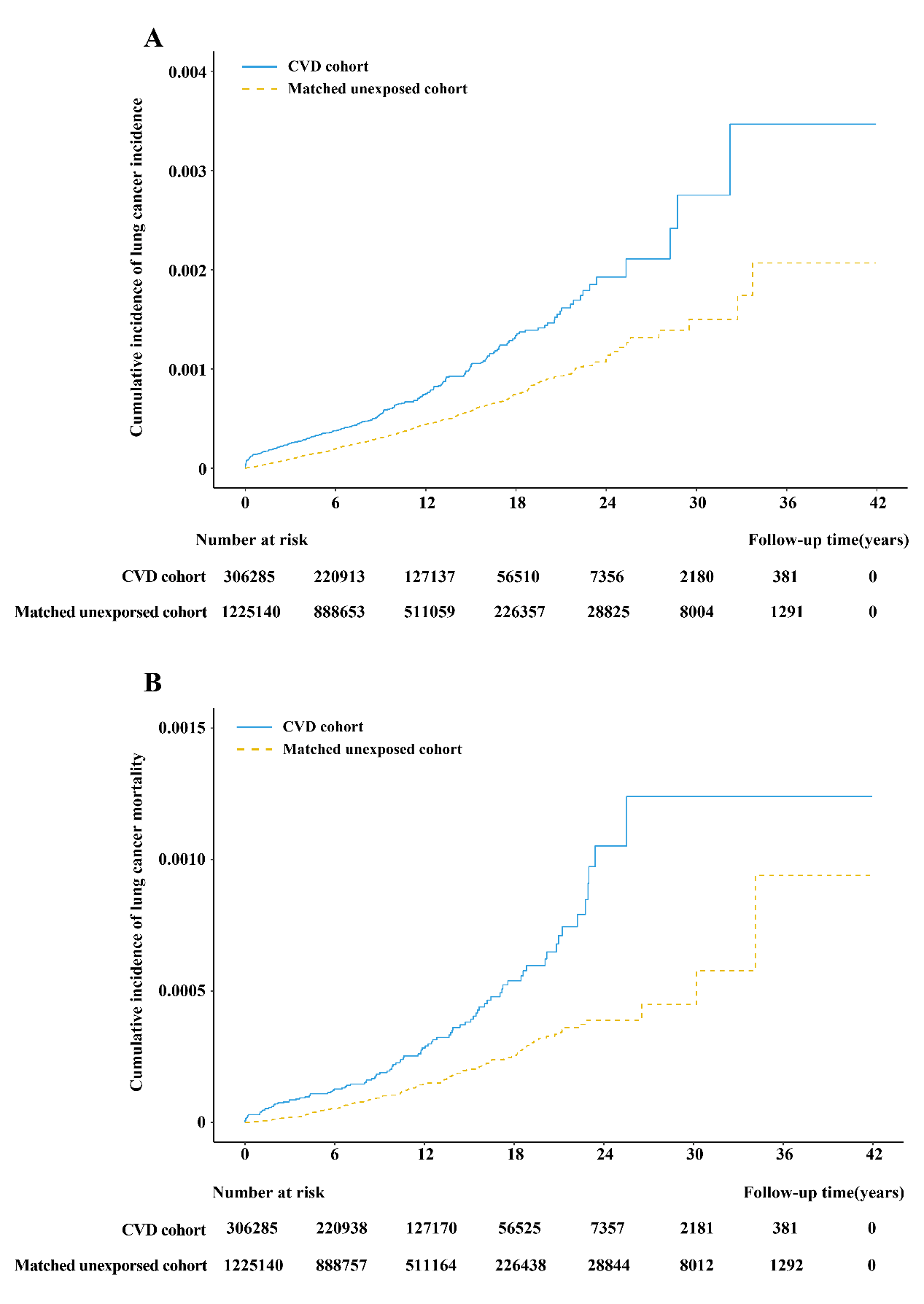


**Figure S2 Cumulative incidence of lung cancer incidence and mortality in CVD cohort (exposed) and matched unexposed cohort**
